# A case report: Rectal endometriosis mimicking rectal cancer

**DOI:** 10.1016/j.ijscr.2018.10.021

**Published:** 2018-10-24

**Authors:** Li-Juan Zhao, Yuan-He Wang, Jing-Dong Zhang

**Affiliations:** Department of Gastrointestinal Medical Oncology, Cancer Hospital of China Medical University, Liaoning Cancer Hospital & Institute, Shenyang 110042, Liaoning Province, PR China

**Keywords:** Case report, Rectal endometriosis, Rectal cancer, Differential diagnosis

## Abstract

Introduction: Rectal endometriosis is rare in women and imaging characteristics are similar with that of rectal cancer, which is one of the most common malignancies.

Presentation of case: A 36 years old woman with a suspicious diagnosis of cervical carcinoma in a tertiary hospital visited our hospital, complaining about vaginal bleeding after copulation for six months, accompanying with constipation and diameter-thinning stool. Vaginal and cervical biopsy only showed chronic inflammation. Colonoscopy found a mass at the rectum 4 cm from the anus, but the biopsy showed different diagnoses. Partial resection was eventually operated and the final diagnosis was confirmed as rectal endometriosis.

Discussion: Rectal endometriosis is prone to be misdiagnosed as rectal cancer. Small specimen is sometimes insufficient to make a correct diagnosis. Extensive examination should be done to confirm the diagnosis and rash decision should never be encouraging.

Conclusion: Rectal endometriosis should always be considered as one of the differential diagnoses in female who have a mass at the rectum. An adequate specimen should be obtained to confirm the histopathological diagnosis.

## Highlights

• Rectal endometriosis and rectal cancer share many common Imaging characteristics.
• Small specimen is sometimes insufficient to make a correct diagnosis.
• Doctors should always pay attention on differential diagnoses.

## 1 Introduction

Endometriosis is a chronic and benign gynecologic disease, with endometrial glands and stroma presenting outside the uterine cavity [1,2]. Endometriosis affects approximately 3%–15% of childbearing age women [1,3,4]. Endometrial tissue can present anywhere of a woman body and can be divided into intra-peritoneal and extra-peritoneal endometriosis according to the anatomic location. The most aberrant places are the ovaries, uterosacral ligaments, large ligaments and pelvic peritoneum. Gastrointestinal tract, lungs, pleura, urinary system, skin, abdominal scar and brain are rare [3,[5], [6], [7]]. Here, we report a case of rectal endometriosis localized at the rectum that was almost misdiagnosed and treated as rectal cancer.

The work has been done in line with the SCARE criteria [8].

## 2 Case report

A 36 years old woman with a suspicious diagnosis of cervical carcinoma in a tertiary hospital several days before visited our hospital. The patient had a regular menstrual cycle. Her childbearing history was G4P1A3, and she gave the birth by cesarean section in 2005. She was diagnosed with hypertension for five years and took medicine regularly, with blood pressure being controlled in normal condition.

**Fig. 1:**
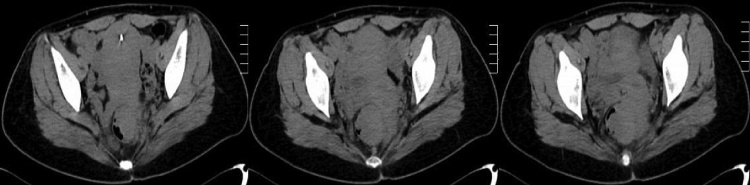
CT showed the rectal occupying lesions.

She complained about vaginal bleeding after copulation for Six months, accompanying with constipation, diameter-thinning stool and an increase of vaginal discharge. She also complained that she had a mild pain sometimes in the low back and pelvis, without obvious association with menstrual cycle. So she came to a tertiary hospital specialized in gynaecology and did some examination according to the doctor’s advice. Pelvic ultrasound showed a 6.0 × 4.3 cm hysteromyoma and a 4.7 × 3.9 cm posterior cervical mass, with the ovaries found no abnormality. Enhanced pelvic computed tomography (CT) showed a 3.5 × 3.9 cm vaginal occupying lesion invading the rectum (Fig. 1) and a 5.8 × 4.7 × 5.3 cm hysteromyoma. The doctor gave a suspicious diagnosis of cervical carcinoma.

**Fig. 2:**
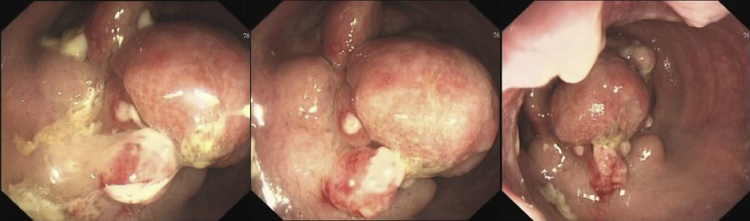
colonoscopy showed the rectal endometriosis mimicking a rectal carcinoma.

**Fig. 3:**
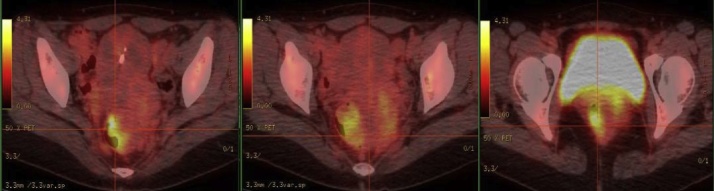
PET-CT showed 18FDG uptake of the rectal endometriosis.

Then the patient came to our hospital for further diagnosis and treatment. Additional examination was done. Examination of tumor markers showed that the serum level of CA125 was 87.9U/ml, 2.5 times of the normal upper limit, with squamous cell carcinoma antigen (SCC), CEA and CA199 within the range of normal value. Physical exam found that cervix uterus was thickened, with irregular shape and locally Protruding nodules. Vaginal and cervical biopsy only showed chronic inflammation. Colonoscopy examination found a mass at the rectum 4 cm from the anus (Fig. 2) and biopsy indicated spindle-cell-like mesenchyma-derived tumor, with the immunohistochemical result not supporting the diagnosis of interstitialoma. Supplementary PET-CT examination showed an increase of 18FDG uptake of the rectal mass (Fig. 3), with the standardized uptake value(SUV) as 4.7. PET-CT reported that it was a submucous tumor, with interstitialoma being suspicious. Then another biopsy examination of the rectum mass was done and the diagnosis result was rectal endometriosis supported by the immunohistochemical result.

Multidisciplinary team, including oncologists, surgical specialists, gynecologists and pathologists, was established to discuss the complex condition. The pathologist supported the diagnosis of rectal endometriosis according to the immunohistochemical result. All doctors shared a common thought that there is a possibility that both rectal carcinoma and rectal endometriosis were existent at the same time, taking the small insufficient biopsy specimen into account. If a radical operation was performed, treating it as a malignant tumor, considering that the rectal mass was only 4 cm away from the anal verge, the patient could not save the anal function, and colostomy would be existent permanently, reducing the quality of life. After a heated discussion, a consensus that partial resection should be operated to confirm the histopathological diagnosis and then to determine the further treatment was achieved. Eventually, the final diagnosis was confirmed as rectal endometriosis and gynecological management was suggested.

Thereafter, the patient was discharged, seeking medical treatment in a tertiary hospital specializing in Gynecology. Gonadotrophin-releasing hormone antagonists was injected subcutaneously once per month for six months, with all the symptoms gradually disappeared. Then, a ring that slowly releases levonorgestrel was put into the uterine cavity, and it should be used until menopause according to the instruction.

## 3 Discussion

Pelvic pain, infertility, dysmenorrhea and dyspareunia are the most common symptoms of endometriosis [3,[9], [10], [11]]. The incidence rate of gastrointestinal endometriosis is 3%–37% of all cases, and rectum and sigmoid colon are the most common sites of it, accounting for 50%–70%. Patients with gastrointestinal endometriosis may present with symptoms such as colic pain, nausea, vomiting, constipation, diarrhea, rectal bleeding, tenesmus, pain during defecation and so on [2,[4], [5], [6],^10^,[12], [13], [14]]. The incidence rate of abdominal scar endometriosis is 0.03%–12%. Most patients with abdominal scar endometriosis had a history of obstetric or gynecologic procedure, and the main clinical manifestations were nodule pain and the sensation of a mass [7,15]. Thoracic endometriosis is rare and can be present with characteristics such as catamenial pneumothorax, haemothorax, haemoptysis and pulmonary nodules [16,17].

The pathogenesis of endometriosis is still unclear. Several hypotheses have been established to explain the development of endometriosis, including retrograde menstruation and ectopic transplantation, coelomic metaplasia, iatrogenic injury, autoimmunity, embryonic theory and stem cell origin, genetic predisposition, hormones and others [3,9,18,19]. An increased level of Interleukin-1, Interleukin-2, tumor necrosis factor and prostaglandins have been found in the endometrial lesions [3]. CA125, CA199, Carbonic anhydrase (CA) Antibody, endometrial Antibody(EMAb) can be used to help diagnose and monitor the development of the endometriosis. CA199 is less sensitive than CA125 [5,6,10]. Imaging examinations of ultrasound, CT and magnetic resonance imaging (MRI) can provide information about the location, size and extent of the lesion [5,10,12]. For rectum and sigmoid colon endometriosis, transvaginal ultrasound (TVUS), transrectal ultrasound (TRUS) and pelvic MRI can also provide information about depth of infiltration in the intestinal wall, percentage of the intestinal circumference, distance between intestinal lesions and the anal verge [20].

Carcinoma, diverticulitis, appendicitis, Crohn’s disease, tuboovarian abscess, ulcerative colitis, irritable bowel syndrome and lymphoma, especially carcinoma, should be considered as differential diagnoses, for they share many clinical symptoms in common but the treatment is quite different [10]. Laparoscopic and pathological examination is the gold standard for the confirmed diagnosis of endometriosis [1,9].

The treatments include medical management and surgeries. For medical management, medications used in endometriosis are hormones, including contraceptives, gonadotrophin-releasing hormone agonists/antagonists and selective progesterone receptor modulators, and non-hormonal drugs such as NSAIDS, aromatase inhibitors and danazol. New therapies such as antiangiogenic factors, statins, TNF-α blockers, PPAR-r and pentoxifylline are being researched. Operation should be designed according to the location, extent and the purpose of the surgery. For intestinal endometriosis, recto-vaginal fistula and anastomotic dehiscence/leak are the most serious postoperative complications, and being less than 10 cm away from the anal verge is an independent risk factor for this [1,3,9,11,20,21].

There is also a problem about recurrence, around 5–20% annually, and studies found

that the recurrence rate after surgical removal of endometriosis of abdominal scar is 1.5%–9.1%. Combination of medical and surgical therapies can be better for some cases to treat the disease, relief pain, improve infertility and reduce the recurrence rate [1,9,21].

## 4 Conclusion

Intestinal endometriosis should always be considered as one of the differential diagnoses in female and reproductive age patients who present with gastrointestinal mass, especially when the mass was located at the rectum and sigmoid colon. Histopathological result of an adequate biopsy specimen should be performed to confirm the diagnosis and then guide the treatment, for rectum and sigmoid colon endometriosis can be cured by medication, therefore, avoiding improper radical surgeries which may induce loss of the anal function, a permanent colostomy and the reduce of the quality of life.

## Conflicts of interest

None.

## Funding

None.

## Ethical approval

Approval has been given by ethics committee of liaoning cancer hospital and institute.

## Consent

Written informed consent was obtained from the patient for publication of this case report and accompanying images.

## Author contribution

Zhang JD - designing the report; Zhao LJ- collecting the patient’s clinical data and writing the paper. Wang YH - Data review and editing.

## Registration of research studies

None.

## Guarantor

Zhang JD.

## Provenance and peer review

Not commissioned, externally peer reviewed.
